# Worlds that collide: conservation applications of behaviour and culture in human–wildlife interactions

**DOI:** 10.1098/rstb.2024.0137

**Published:** 2025-05-01

**Authors:** Estelle Meaux, Culum Brown, Sarah L. Mesnick, Caitlin O'Connell-Rodwell, Hannah S. Mumby

**Affiliations:** ^1^ Applied Behaviour and Ecology Group, School of Biological Sciences, The University of Hong Kong, Hong Kong; ^2^ CNRS, EthoS (Éthologie Animale et Humaine) - UMR 6552, Université de Rennes, Université de Normandie, Rennes 35700, France; ^3^ School of Natural Sciences, Macquarie University, Sydney, New South Wales NSW 2109, Australia; ^4^ Southwest Fisheries Science Center,, National Marine Fisheries Service, National Oceanic and Atmospheric Administration, La Jolla, CA 92037-1508, USA; ^5^ Department of Biology (Center for Conservation Biology), Stanford University, Stanford, CA 94305., USA; ^6^ Department of Politics and Public Administration, The University of Hong Kong, Hong Kong, Hong Kong

**Keywords:** biodiversity conservation, local communities, wildlife management, behaviour change theory

## Abstract

The behaviour of both humans and wildlife is central to the conservation of biodiversity because conservation requires human actions at multiple scales. In species with evidence of socially learned behaviour and culture, the juxtaposition of human and animal culture increases the complexity of human–wildlife interactions and their investigation but also offers opportunities to mitigate negative interactions. In this paper, we consider the language used to analyse human–animal interactions and we review the effect of culture and socially learned behaviours on those interactions. We investigate how knowledge of culture and theory from behavioural studies can be used to negotiate the complex interactions between humans and wildlife, providing specific examples of how culture can be mined for developing policies regarding negative interactions. We highlight that interactions between animal and human culture are central to the conservation of wildlife, and that such human–wildlife interactions are a key target for studies of biodiversity conservation. Integrating culture and social learning into conservation research offers scope to leverage knowledge gaps, misconceptions and concerns into conservation actions that are targeted, relevant and meaningful.

This article is part of the theme issue ‘Animal culture: conservation in a changing world’.

## 1. Introduction

The widespread impacts of human activities on nature highlight the importance of human behaviour in understanding and addressing biodiversity conservation [1,2]. Whether a single individual, a local community or governmental agencies, humans are the decision-makers and the drivers of conservation [3]. At the same time, humans are also the main cause of the loss of biodiversity, precipitating the necessity of conservation [1]. Alongside this, wildlife behaviour can be viewed both in terms of a vital part of biodiversity to be conserved, and an important factor to consider in taking actions to conserve biodiversity [2,4]. The behaviours of humans and wildlife are often inextricably intertwined because they interact and change in relation to each other, allowing the emergence of a cultural coevolution [5]. Therefore, the phrase ‘conservation means behavior’ as coined by Schultz [2] takes on multiple meanings from both human and non-human perspectives, which include behaviour across species representing the cause, impetus and object of conservation.

Understanding the role played by cultural and socially learned behaviours is central to conservation because these behaviours are required to conserve the evolutionary potential of wildlife populations [6]. Scholars have emphasized that this evolutionary potential extends far beyond possible changes in functional genes and includes other processes of inheritance, including social learning and culture-based phenotypic diversity [7]. The behaviour of non-human animals (hereafter shortened to ‘animals’) changes in response to human behaviours, and elements of this may become cultural change. A classic example is deforestation in Israel, which made black rats (*Rattus rattus*) relocate from oak forests to pine forests, where new cultural behaviours emerged to feed on pine seeds [8]. Here, we define culture as behaviours shared by members of a group or a community through socially learned and transmitted information [9,10]. Their transmission can occur between parents and offspring (vertical transmission) and through different social groups within the same generation (horizontal transmission) [11]. To maintain the evolutionary potential and long-term persistence of populations, it is important to consider cultural variation as a crucial element of biodiversity to be preserved [12]. However, culture induces unique behaviours in sub-populations of animals, which may be caused by or impact their interactions with humans.

Human–wildlife interactions (hereafter ‘HWI’), by definition, involve both human and wildlife behaviour and their relationship. HWI also often represent challenges to conservation goals, and a better understanding of the underlying dynamics is crucial to building holistic and effective approaches for addressing them. Here we consider HWI broadly, encompassing both negative, neutral and positive interactions [13]. We note that different terminology is used to describe the various types of HWI. ‘Co-occurrence’ or ‘cohabitation’ are mostly used for neutral interactions and emphasize humans and wildlife being in the same space, while ‘co-existence’ may imply sharing a space in a way perceived to be peaceful; ‘co-adaptations’ and ‘cooperation’, acting together, emphasize the positive dimensions or instances of HWI [14]. Negative interactions can be labelled as ‘conflict’, which emphasizes negative impacts, yet this framing is still critically debated, including discussion of whether language perceived as negative overemphasizes or reinforces antagonistic interactions [15,16]. Here we choose to use the term HWI, while acknowledging those who have experienced negative interactions with wildlife. We do not intend to diminish or minimize those lived experiences through our use of neutral language. Moreover, the language also varies distinctly between academic discourse and practice. A holistic approach could help to address this potential barrier separating the terms adopted by scholars in their research from terminology used in policies by practitioners or used by local communities to describe their lived experience [16].

This review builds on the discussion and recommendations of the working group on HWI at past meetings of the UN Environment Program Convention on Migratory Species expert working group on animal culture and social complexity [17–19]. We aim to demonstrate the importance of considering the cultural and social aspects of HWI as a key target for conservation research and highlight how social learning, culture and other aspects of sociality are important when planning, implementing and studying actions for the conservation of species involved in HWI. We propose a holistic approach for integrating behavioural and cultural aspects of HWI into conservation decisions. We describe the effects of culture on HWI by analysing how animal and human cultures interact and influence each other, and how such cultural interactions can lead to novel opportunities for research on mitigation strategies and conservation management. We also propose general recommendations for mitigating negative HWI with the overarching aim of maintaining cultural behaviours, since a change in cultural behaviour may threaten the conservation status of wildlife groups or populations.

## 2. The effects of culture on human–wildlife interactions

The current gap in understanding of the complex interactions between human and animal culture can confound conservation efforts and even contradict intended management objectives. Those interactions between human and animal cultures often have population-level consequences and can generate conservation impacts in two senses: first, when animal behaviour generates impacts on human activity and second, when human activities generate impacts on animal populations. The examples cited below are summarized in table 1, which highlights the social learning involved in the animal and its impacts on HWI, and whether those impacts are positive, negative or both. In some of these examples, social learning and culture are not directly discussed in the original publication, but they may be important components of the behaviour and HWI described.

**Table 1.** Examples of published studies highlighting the role of culture and socially learned behaviours in human–wildlife interactions.

animal social learning	animals (species and common names)	directionality of impact	human activities	description of social learning	impacts	references
**foraging techniques using human food sources**	African elephant *Loxodonta africana*	animal > human	agriculture (cultivated food)	crop raiding and the diverse techniques to counter the different current mitigation solutions	negative *economic income* *human safety*	[20–22]
	chimpanzee *Pan troglodytes*	animal > human	agriculture (cultivated food)	process of selection in the agricultural crop to raid	negative *economic income*	[23]
	sperm whale *Physeter macrocephalus*	animal > human	commercial fishing	adult males learn to depredate catch from fishing gear	negative *economic income*	[24]
	wolf *Canis lupus*	human > animal > human	agriculture (livestock)	unselective culling of the wolves resulted in an increase in breeding pairs owing to the fragmentation of the social hierarchy of the territorial groups, which ultimately increased livestock depredation	negative *economic income* *potentially negative human safety*	[25]
	tits *Parus major*	human > animal > human	milk consumption	special technique to open milk bottles	negative (minor) *household income*	[26,27]
**other food-related behaviours**	sulfur-crested cockatoo *Cacatua galerita*	animal > human	garbage production	special technique to open household garbage bins	negative (minor) *living and sanitary conditions*	[28]
	honeyguide *Indicator indicator*	cooperation	traditional food-gathering behaviour	recognizing human acoustic cues, then guiding them to a natural beehive	positive *localization of the hive to access honey for humans* *access to beeswax for animals*	[29]
	bottlenose dolphin *Tursiops truncatus*	cooperation	traditional fishing	synchronizing hunting movements with human techniques	positive *facilitates fishing for both*	[30]
	killer whale *Orcinus orca*	cooperation	traditional baleen whale (parvorder *Mysticeti*) hunting	guiding humans to the location of the baleen whale, then helping in hunting it	positive *localization of the baleen whale for humans* *access to baleen meat for animals*	[31]
**food-related behaviours**	blue whale *Balaenoptera musculus*	human > animal	shipping transportation	foraging locations transmitted through generations	negative *increases mortality owing to ship strikes*	[32]
	colonial vultures *Gyps* spp*.*	human > animal	reintroduction programme	general foraging behaviours	positive *location of feeding stations is based on the social transfer of foraging behaviours*	[33,34]
**migratory behaviours**	migratory species	human > animal	infrastructure (roads, fences and dams)	migration trajectories transmitted through generations	negative *blocks migration pathways*	[35]
**general behaviours depending on social learning**	African elephant *Loxodonta africana*	human > animal	selective poaching (larger animals, often corresponding to the older ones)	global social transmission (older individuals represent knowledge repositories)	negative *removal of cultural key individuals may disrupt the cultural transmission*	[36]
	small cetacean species living in fission–fusion society (e.g. dolphins)	human > animal	commercial fishing (release of individuals unintentionally captured)	global social transmission (e.g. survival, reproduction…)	negative *stress and injuries may disrupt the social network*	[37]

### (a) Impact of socially learned animal behaviour on human activities

The influence of animal culture on human lives is a vital conservation consideration. Traditionally, research and practice in the field of HWI have focused on negative interactions, commonly referred to as conflicts, for cases requiring balance between resource demands of humans and wildlife [16]. This includes, for example, depredation of commercial fisheries and livestock and crop foraging, and it involves diverse taxa [13]. Beyond the physical impacts that such negative HWI can represent, they also usually include a local negative economic impact, which does not encourage an interest in their conservation *in situ* [38]. For example, group members can learn special techniques or locations from groupmates to forage from human food sources (table 1). The interactions between humans and other species can also be positive, providing mutual benefits through cooperation [31]. Such interactions involve intentional behaviours directed towards a goal shared by participants from both species, such as accessing a specific food type. One example is the cooperative interaction between humans and honeyguides (*Indicator indicator*) to retrieve food produced by honeybees in Africa (table 1). Although it is clear that human cultural traditions play an essential role in the maintenance of this mutualistic interaction, it remains to be confirmed whether honeyguides use social learning to spread this behaviour to conspecifics [39]. A similar interaction involves fishers cooperating with dolphins (table 1), although here, there are signs suggesting a cultural transmission of these behaviours within the dolphins’ populations, with a separation between the groups cooperating with humans for fish hunting and those that do not [40].

### (b) Anthropogenic effects on animal culture and sociality

Cultural behaviours of wildlife species may potentially increase their vulnerability to human-caused changes in their environment. Although human activities can impact animal lives in many ways, some populations may be more affected than others, since cultural traditions are often site-specific. For example, the simple behaviour of avoiding humans or their activities directly modifies the habitat use of some animal populations [41]. Human activities can disrupt socially learned migratory behaviours, for example, with the use of infrastructures that can block migration pathways (table 1). Even in oceans, the increase in anthropogenic activities over the past decades has heavily influenced the habitat and subsequently the lives of marine migratory species (table 1).

The disruption of social structure in wildlife populations can also have consequences for information transfer and ultimately for survival and reproduction. Sub-lethal human impacts can also disrupt animal social networks and the transmission of information. Many dolphin species live in fission–fusion societies whereby survival and reproductive success depend on social learning [42], but they are often under pressure from the fishing industry. Even when individuals are released alive after capture, the stress and potential injuries provoked by bycatch events may have a significant impact on the social network of the whole community (table 1). This effect may also occur in some fish species, where culture and social learning have also been demonstrated [43].

### (c) Conservation applications of cultural interactions between humans and animals

Though culture can sometimes be considered as maladaptive to environmental change [44], it should not be assumed that culturally determined behaviours are inflexible and inevitably lead to vulnerability. The process of social learning can improve the fitness of individuals by enhancing adaptive behaviours, which in turn increases the population's viability and resilience. Anthropogenic activities can, for example, lead to new foraging opportunities and innovation that rapidly spread through different social groups of conspecifics, with the classic example of the milk-bottle-opening behaviour of tits (table 1). Interestingly, such innovative techniques are not fixed in time but can quickly adapt to new environmental challenges [45]. Another case where cultural behaviours can increase adaptivity to the environment is when wildlife species are confronted with urbanization. For instance, sulfur-crested cockatoos developed techniques to open household garbage bins in the city of Sydney, Australia, making geographical groups culturally distinct from each other (table 1). The emergence and transmission of innovations through social learning can enable wildlife species not only to benefit from new food resources but also to adapt to environmental change.

The study of cultural traits and socially learned behaviours can add essential information when assessing existing and potentially negative HWI, and whether there will be threats to conservation [46]. However, HWI can be perceived differently depending on the organismic guilds, based on psychological factors [47]. Regardless of the species or taxa, improving our understanding of the underlying animal information network and evaluating how problematic behaviour may be transferred between conspecifics can help in the development of mitigation strategies. In the reintroduction programmes for vultures, it is common to set up feeding stations to help restore populations, and researchers have emphasized the importance of considering the social transfer of foraging behaviours between individuals in deciding on the location of such feeding stations with the aim of restoring their social networks as well (table 1).

Throughout history, animal species have inspired humans in their traditions, arts, rituals, religious and spiritual beliefs [48]. This significance gave such species the status of ‘cultural keystone species’ [49]; for example, in some Pacific Islands, local communities considered certain shark species as ‘ancestral gods’ [50]. Such perceptions of wildlife by humans can affect actions taken by locals in the face of negative HWI. For instance, people have feared large predators over millennia, while still valuing their presence in the landscape. Felids are known to occasionally prey on humans, but only in some populations, which suggests that this behaviour is a localized culture, and the image of big cats perceived by human communities that are preyed upon consequently differs from other local communities [51]. This ‘cultural coevolution’ [5] between humans and wild animals leads both parties to modify their cultural behaviours to coexist in a shared landscape.

A more recent trend is that of wildlife tourism, which can also have a significant impact on animal culture. One of the many examples of this is the feeding of wildlife, which often modifies animal movements within their territories, as is, for example, the case with sharks [52,53]. Moreover, human lifestyles quickly change, with subsequent changes in the cultural behaviours related to HWI. In the example of human–dolphin cooperation mentioned earlier, the economic context influences fishers to use different methods for better yields, thus decreasing their tendency to cooperate with dolphins [54]. In the case of human–honeyguide cooperation, the spread of apiculture has progressively replaced the honey-hunting tradition, reducing the transmission of the cooperative behaviours with honeyguides [55].

## 3. Recommendations

### (a) Integration of animal and human cultural knowledge in conservation measures

Culture is an important aspect of biology that should be considered within existing conservation outcomes when managing populations that are impacted by conflictual interactions with humans. Many HWIs are in a constant process of change, with animals continually and reciprocally adapting their behaviour to each novel mitigation solution implemented by humans. For example, in wild populations of sulfur-crested cockatoos inhabiting urban areas, birds that have learned socially to forage in the residential trash bins are constantly challenging the techniques that humans use to protect their bins—techniques that are themselves passed along socially [56]. This is an example of the recent concept of ‘co-culture’, defined as cultures in distinct animal populations—human/non-human, inter/intraspecies—that coevolve by influencing the culture of each other [57]. Such cases of ‘cumulative cultural arms races’ between humans and wild species indicate that conservationists need to be able to develop multiple options to manage HWI instead of focusing on just one or two mitigation solutions [58].

To preserve biodiversity, it is essential to include the collection of empirical evidence of cultural diversity, social learning networks and migratory behaviour connections, and to conserve cultural repositories and capacities, with the aim of integrating them into the development of conservation and management strategies. This includes developing and maintaining methods to facilitate the assessment of cultural knowledge and social transmission in the animal groups involved in HWI [9]. For example, camera traps and drones could be used to extend the collection of behavioural data and to infer animal social networks [59,60]. Another example is to use acoustic monitoring to analyse vocal culture in birds, which can help to discriminate geographical groups based on the variation in their vocal behaviours [61]. Novel methods can also be developed to solve negative HWI based on knowledge of animal behaviour. This has been recently shown with the use of translocated scents to modify the territorial movements of African wild dogs (*Lycaon pictus*) [62].

Raising the awareness of local human communities to the presence of culture in animal populations involved in HWI may be a helpful strategy to inspire conservation [38]. It can also be used to enhance the understanding of the concept that culture involves animals socially learning certain types of behaviours, resulting in changes to HWI that evolve over time. This, in turn, means that finding solutions to reduce negative interactions is a moving target, necessitating a toolbox of mitigation measures [17] and a proactive approach that targets mitigation measures early, during the ‘window of opportunity’ during which only a few individuals are involved and before a negative behaviour sweeps through an animal population [17,24]. Emphasizing how culture is important in some socially complex species can also reveal to local human communities and wildlife managers the importance of specific individuals that act as repositories of knowledge, and that an unselective killing of individuals may result in a high disturbance of the social networks and information transfer. For example, in African elephants, the effects of the removal of older individuals by culling can have long-term consequences, whereby the population lacks social and environmental information that they would usually have learned from these older group members [63]. This highlights the importance of not only age but also of social characteristics of certain elder individuals that either seek out or choose to spend time with younger individuals, making it easier to transfer knowledge and potentially specific behaviours [64].

To mitigate negative HWI, it is essential that all those involved are engaged in co-developing conservation solutions, and that efficient communication is established between them at all stages of implementation. Hence, understanding and respect for the local culture of each person or group involved, and specifically for the human communities facing the direct impacts, e.g. farmers and fishers, is essential for managing, mitigating and reducing the negative effects of HWI [38]. To address negative HWI, there is a need to integrate culture from both animals and humans in communication and education efforts focused on the conservation and HWI.

### (b) The applications of behaviour change theory to the cultural aspects of HWI

The knowledge that humans have of animal culture and behaviour, in general, can be utilized to optimize conservation measures. Connecting our own cultural behaviour with our knowledge of animal culture can help us to adopt a holistic approach to the mitigation of HWI and the conservation of the species involved. Methods relating to behavioural change have been highlighted as essential to conservation interventions [2,65,66], and we emphasize here their importance in managing HWI.

When behaviour change theory is applied to humans, it is well established that normative behaviour and social influence are relevant to the design of conservation interventions [67,68], and we previously reviewed the equal relevance of cultural behaviours with the same purpose, yet there has not been much focus on combining both elements and integrating the results into the development of conservation policies around HWI. Behaviour change methods can impact different scales of the problem. Travers *et al*. [66] identified several main actions to make conservation behaviour change more effective, including that practitioners should base their actions on humans’ social, cultural, psychological and cognitive behaviour. If we take the case of conservation campaigns, research has shown that strategies based on behaviour change theory can be efficient in raising attention and awareness in the general public, and when combined with policies, regulations and education, it positively impacts conservation outcomes [69,70]. This suggests that changing the human cultural traditions underlying HWI may require a different approach from the traditional campaigns for conservation to target the specific audiences and increase the probability of conservation impact.

Similarly, principles of human behaviour change theory can be applied to animals, specifically when culture plays an important role in how animal populations evolve in their environment [71]. Animal species with socially learned behaviours involved in negative HWI can be resilient to mitigation solutions, owing to their cognitive and social capacities to overcome methods typically used in such solutions (e.g. habituation) [72]. This is the case in elephants, where it is critical to adopt approaches that include the identification of demographic and personality traits of the concerned elephant populations, the hormonal status of the perpetrators (i.e. musth males [73]), an assessment of the cultural behaviours of both elephants and humans that are involved locally and as well as implementing interventions that combine academic and local experts [74]. Based on our knowledge of elephant aversion behaviour for bees, mitigation solutions have included the use of beehive fences [75] or alternatively the uses of dummy beehives [76], aggressive bee sounds [77] and their alarm pheromones [78]. There is also a need for cross-over studies that analyse how human behaviour changes can be used to determine behavioural changes in animals, e.g. using artificial intelligence tools to assess animal behaviour in conservation management [79]. Box 1 summarizes this section by presenting our main recommendations for conservation management of HWI.

Box 1. **Recommendations to integrate culture and social learning in human–wildlife interactions (HWI) for conservation actions.**

- Recognize that it is important to take local human culture and traditional practices into consideration when implementing a mitigation solution. Pre-implementation discussions between all those involved are necessary, as well as post-implementation feedback, to ensure that the solution(s) have been accepted and adopted appropriately by all actors.
- Review how behaviour change methods may be relevant to the mitigation of negative HWIs and how normative behaviour and culture are required for the design of such interventions.
- Recognize that animal cultures have conservation consequences and emphasize communication and education around this topic to improve the attitudes of locals towards animal conservation and management of HWI.
- Develop a database on novel animal behaviours associated with negative HWI. This should cover as wide a range of species as possible and detail evidence of impact. Potential for applications in other contexts or species should also be highlighted.
- Develop methods to analyse the process of social learning of cultural traits in animals, including inferring the transmission of information across animal social networks and how it is modified by human behaviours. This will also help in predicting how HWI can evolve in the long term or to prevent them from turning into a conflictual relationship if actions are implemented at an early stage of the interaction.
- Develop a ‘toolbox’ of mitigation measures for stakeholders to rely on, as opposed to a single solution to negative HWI, therefore enhancing the success of the managing actions.

## 3. Conclusion

We highlight the value of considering the interactions between wildlife and humans through the lenses of culture and socially learned behaviour. We emphasize the importance of the integration of both human and animal culture and social complexity into conservation actions associated with HWI. By including examples showcasing the opportunities to develop new insights as well as new solutions to the issues brought by negative HWI, we show the need to address knowledge gaps, misconceptions and concerns. Furthermore, being thoughtful about our use of language and the construction of terminology framing HWI can offer a different perspective to the present problems brought on by negative interactions. This includes both the perception of wildlife as a threat and the opportunities for coexistence in shared landscapes. Conservation actions in the management of HWI can be tailored by considering site-specific cultural behaviours and in doing so, these actions can inform our understanding of the role of culture and socially learned behaviour in HWI. The discoveries of cultural behaviours in species (including invertebrates) constantly increase [80], and with the equally increasing use of new technologies for wildlife observation [81], it may be possible to rapidly adapt our approach to conservation and HWI by including cultural aspects of both humans and wildlife.

## Data Availability

This article has no additional data.
